# Chromatin Accessibility Data Sets Show Bias Due to Sequence Specificity of the DNase I Enzyme

**DOI:** 10.1371/journal.pone.0069853

**Published:** 2013-07-26

**Authors:** Hashem Koohy, Thomas A. Down, Tim J. Hubbard

**Affiliations:** 1 Wellcome Trust Sanger Institute, Hinxton, Cambridge, United Kingdom; 2 Gurdon Institute and Department of Genetics, University of Cambridge, Cambridge, United Kingdom; National Institutes of Health, United States of America

## Abstract

**Background:**

DNase I is an enzyme which cuts duplex DNA at a rate that depends strongly upon its chromatin environment. In combination with high-throughput sequencing (HTS) technology, it can be used to infer genome-wide landscapes of open chromatin regions. Using this technology, systematic identification of hundreds of thousands of DNase I hypersensitive sites (DHS) per cell type has been possible, and this in turn has helped to precisely delineate genomic regulatory compartments. However, to date there has been relatively little investigation into possible biases affecting this data.

**Results:**

We report a significant degree of sequence preference spanning sites cut by DNase I in a number of published data sets. The two major protocols in current use each show a different pattern, but for a given protocol the pattern of sequence specificity seems to be quite consistent. The patterns are substantially different from biases seen in other types of HTS data sets, and in some cases the most constrained position lies outside the sequenced fragment, implying that this constraint must relate to the digestion process rather than events occurring during library preparation or sequencing.

**Conclusions:**

DNase I is a sequence-specific enzyme, with a specificity that may depend on experimental conditions. This sequence specificity is not taken into account by existing pipelines for identifying open chromatin regions. Care must be taken when interpreting DNase I results, especially when looking at the precise locations of the reads. Future studies may be able to improve the sensitivity and precision of chromatin state measurement by compensating for sequence bias.

## Background

The development of animals from zygotes to adults and the differentiation of cells into tissues and organs depends on intricate programs of cell-type and stage-specific transcriptional regulation. This is accomplished by complex interactions between DNA sequence and transcription factors (TFs) at regulatory elements including enhancers, promoters, silencers, and insulators. Equally importantly, nucleosome positioning, histone modifications and DNA methylation can modify the function of these elements, for instance by modulating the accessibility of the DNA to TFs. Therefore, to understand regulatory mechanisms, it is important to be able to assess chromatin state.

DNase I is an endonuclease which digests double-stranded DNA. It is expressed widely in humans and other animals and naturally functions as a waste-management nuclease [1]. It may also play a role in the destruction of DNA during some forms of cell death [1]. But it can also be used in the laboratory as a probe for protein-DNA interactions. DNase hypersensitivity assays use DNase I to digest preparations of whole chromatin, with certain regions – corresponding to open chromatin – digested with much greater efficiency. A complementary technique, called footprinting, relies on the protective effects of proteins binding DNA to identify transcription factor binding sites, potentially at base-pair resolution [2]. This paper focuses on hypersensitivity assays, although we expect DNase I used in footprinting experiments to behave similarly.

Hypersensitivity assays were originally developed in the 1970s, using Southern blots as the readout to measure the DNase sensitivity of targeted regions [3]. While these experiments offered some important early insights into gene regulation, the assays were labour-intensive and low throughput. More recently, the technique has been scaled up, firstly to parallel assays of many genomic regions on a microarray [4], [5], and then to truly genome-wide studies using high-throughput sequencing platforms for readout [6]–[10].

A typical DNase-seq experiment comprises the extraction of chromatin from cells under mild conditions, digestion using a carefully controlled amount of DNase I, then – following removal of protein – a size-selection step (for instance, using a sucrose gradient) to pick short fragments, *i.e.* those derived from regions where two cuts have occurred in fairly close proximity. These fragments are end-repaired (DNase I sometimes leaves a 1bp overhang), linked to sequencing adaptors, amplified and sequenced, generating short sequence tags which identify one end of a selected fragment. After mapping to the genome sequence, tools such as Hotspot [8], [11] or F-Seq [12] are used to identify regions with many such tags, and consequently a high density of DNase I cut sites.

Sequencing technologies do not behave like idealized models and, over the past few years, several groups have reported sequence composition bias in the reads observed from ChIP-seq and RNA-seq experiments and they have presented different models for normalization of such data[13]–[15]. To the best of our knowledge there has not been any report of sequence bias in DNase-seq data.

In this study, we show significant sequence composition bias in DNase-seq reads and based on our analysis we show that, contrary to previous reports [4], [5], the DNase I enzyme has a substantial degree of sequence preference. We carried out this investigation after observing a strong sequence composition bias close to the boundaries of ENCODE open chromatin region data sets [6]. Our work demonstrates that deriving open chromatin regions from current DNase-seq data, and presumably related DNase-array and DNase-chip technologies, requires extra care. It is necessary for processing algorithms to take sequence bias into account to avoid misinterpretations, for example, reporting false positive open chromatin region calls.

## Results

### Sequence Specificity of DNase I Enzyme

While sequencing platforms used to assay experiments such as DNase-seq would ideally detect and report all fragments with equal efficiency, in practice they often show a degree of compositional bias, typically taking the form of under-representing fragments with the most extreme (highest or lowest) G+C content [14]. Therefore, it is of interest to investigate compositional biases seen in DNase-seq reads.

**Figure 1:**
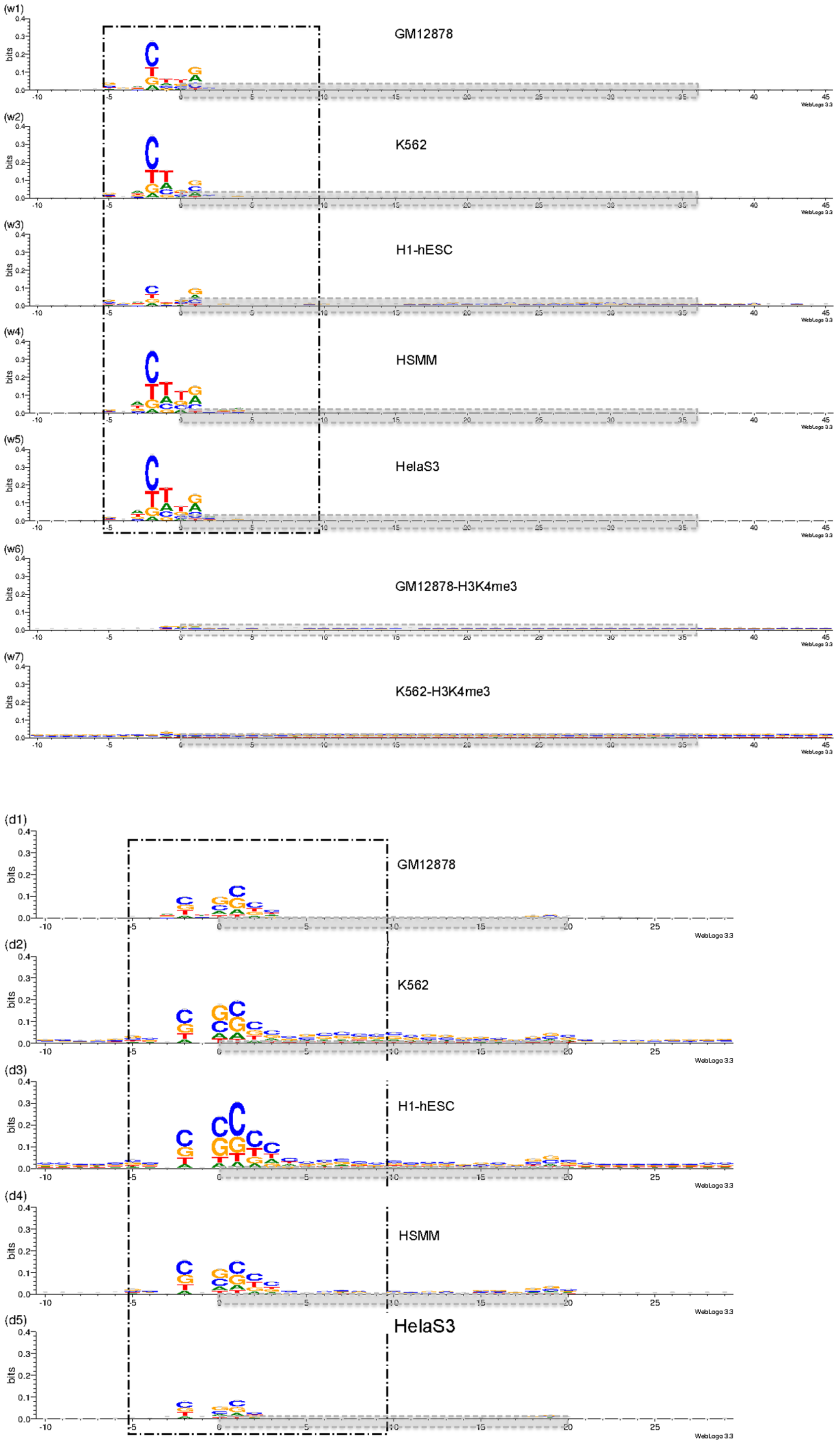
Sequence around the 5′ ends of DNase-seq reads shows substantial bias. Consensus sequence composition around the 5′ ends which are shown as sequence logos representing position-weight matrices. Panels w1–w5 are depicting the pattern from DNase-seq data from UW. Panels w6 and w7 are histone modification ChIP-seq data which are considered as control sets. Panels d1–d5 are illustrating DNase-seq from Duke data. The boxed regions (−5 to +9) were used as position-weight matrices when scoring reads for subsequent analyses. The horizontal boxes, shaded in grey, represent the tag regions that were sequenced in a given experiment.

We studied potential compositional biases in five sets of DNase-seq reads corresponding to GM12878, H1-hESC, K562, HSMM and HeLa-S3 cell lines from both the University of Washington (UW) and Duke University (Duke). These data have been generated as part of the ENCODE project [6], [16]. More details about the accessibility of these data can be seen in Table 1, in which the reader can also see information about other data sets that were used to support our argument. For each of these five cell lines from each of the sources, we extracted all read mapping positions on chromosome 22 (GRCh37/hg19 coordinates) and their corresponding sequences plus 10bps of flanking sequences and built position-weight matrix models of the consensus. In all of the UW libraries we studied (Figure 1:w1–w5), and many of the Duke libraries (Figure 1: d1–d5), the sequence bias across the bodies of the reads was relatively minor. Some, but not all of the Duke libraries showed a significant G+C enrichment across the body of the read (Figure S2), which seems consistent with some of the more extreme amplification biases previously reported [14]. However, we observed a strong specific sequence preference around the 5′ end of the DNase-seq tags. This is noteworthy because the constrained region spans the 5′ end of the tag, and in the majority of the UW libraries, the most constrained position lies outside the sequenced tag. This implies that at least a substantial fraction of the bias we see in these reads could not have been introduced during adaptor-ligation, library amplification or sequencing, since the bases outside the sequenced tag would not have been present at those stages.

**Table 1:** Data used in this study.

	Cell Line	Protocol	GEO	File Name	Reference
Main Data	GM12878	UW	GSE29692	wgEncodeUwDnaseGm12878AlnRep1.bam	[6], [16]
		Duke	GSE32970	wgEncodeOpenChromDnaseGm12878AlnRep1.bam	[6], [16]
	H1-hESC	UW	GSE29692	wgEncodeUwDnaseH1hescAlnRep1.bam	[6], [16]
		Duke	GSE32970	wgEncodeOpenChromDnaseH1hescAlnRep1.bam	[6], [16]
	K562	UW	GSE29692	wgEncodeUwDnaseK562AlnRep1.bam	[6], [16]
		Duke	GSE32970	wgEncodeOpenChromDnaseK562AlnRep1.bam	[6], [16]
	HSMM	UW	GSE29692	wgEncodeUwDnaseHsmmAlnRep1.bam	[6], [16]
		Duke	GSE32970	wgEncodeOpenChromDnaseHsmmAlnRep1.bam	[6], [16]
	HeLa-S3	UW	GSE29692	wgEncodeUwDnaseHelas3AlnRep1.bam	[6], [16]
		Duke	GSE32970	wgEncodeOpenChromDnaseHelas3AlnRep1.bam	[6], [16]
Supporting Data	LNCaP	UW-like	GSM822388	GSM822388-lncap-dht-dnasei-new.bed.gz	[18]
		Duke-like	GSM816637	Duke-DnaseSeq-LNCaP	[19]
	MCF-7	UW-like	GSM822389	GSM822389-mcf-7-v-dnasei-new.bed.gz	[18]
		UW	GSE29692	wgEncodeUwDnaseMcf7AlnRep1.bam	[6], [16]
	HepG2	Duke-like	GSM748507	GSM748507-HepG2-DukeDNaseSeq-align-rep1.bed.gz	[20]
Control Data	GM12878-H3K4me3	UW	GSE35583	wgEncodeUwHistoneGm12878H3k4me3StdAlnRep1.bam	[6]
	K562-H3K4me3	UW	GSM945165	wgEncodeUwHistoneK562H3k04me3StdZnf2c10c5AlnRep1.bam	[6]

This table describes the data used for this study. The main data includes five cell lines from both University of Washington (UW) and Duke University (Duke). Bias from this data has been illustrated in Figure 1 panels w1–w5 and d1–d5. The supporting data rows describes data from four more experiments that either followed the UW or Duke protocols. The bias of these data is illustrated in Figure S1 panels I-III and IV-V. The control data is built from two ChIP-Seq histone modification data sets. We illustrated results of our analysis based on these two data sets in Figure 1 w6–w7. In the protocol column, those data generated in UW and Duke are denoted as UW and Duke respectively, but those which are a slight modification of these protocols are denoted as UW-like and Duke-like. The file names column shows the name of the files that we obtained from the ENCODE repository at UCSC and used in this study.

The robustness of these sequence patterns was evaluated by performing the same analysis for at least one additional replicate from each of the GM12878, K562, HSMM and HeLa-S3 cell lines. The sequence pattern was very consistent across all the UW DNase-seq data sets we considered. The degree of constraint varied a little, but was considerably weaker for H1-hESC compared to the other samples. There were no replicates available for H1-hESC so it is not clear whether there is a biological difference between cell lines leading to a weaker constraint for H1-hESC or if it is due to a technical problem with this particular sample.

The Duke data sets show a broadly similar degree of constraint close to the cut site, but the exact sequence is somewhat different. There is also a small amount of additional constraint at the 3′ end of the tag. One possible explanation could be different conditions for the DNase I digestion step. However, the Duke protocol [17] differs from the UW protocol in that, rather than size-selecting short fragments lying between two DNase I cut sites, it uses a second nuclease to make additional cuts. Specifically, one of the two sequencing adaptors is ligated to the DNase I-cut ends, then the offset-cutting restriction endonuclease MmeI is used to generate short tags to which the second sequencing adaptor can be ligated. While the main recognition sequence for MmeI is present in the first sequencing adaptor, it is possible that there is some additional sequence preference at positions around either the recognition site or the cut site, and this, in addition to DNase I bias, is driving the patterns seen at the boundaries of the Duke reads.

In order to investigate if the observed bias is lab or protocol dependent, we investigated three more data sets (See Table 1 rows named as “Supporting Data”) generated in different labs but following established protocols. The first set we considered comes from [18](Figure S1:I-III). The underlying protocol used to generate DNase I data in this study is a slight modification of the UW protocol with one difference being that short read tags produced are of length 49bp (13bp longer than UW data). The LNCaP cell line data set (both LNCaP data sets) from this study showed a similar bias to that observed in the UW data.

The other two data sets we looked at were LNCaP from [19] and HepG2 cell lines [20] (Figure S1:IV-V). These studies both used the Duke protocol and showed a similar pattern of sequence bias to that observed in the Duke data. To the best of our knowledge, all currently published data sets follow one of these two protocols. As a control we inspected ChIP-seq reads from the same cell lines (for two examples see Figure 1:w6–w7) and saw no comparable sequence preference around the 5′ end of the tags, offering further evidence that the bias is not a feature of the generic library preparation, sequencing, or read-mapping processes. From this we conclude that all existing DNase I short read tag data sets are likely to suffer from one or the other pattern of bias.

Given the precise alignment of the preferred sequence motif to the sequence tag ends, it seems unlikely that this motif is a general feature of open chromatin (see Figure S3). We therefore conclude that this sequence motif represents a sequence preference inherent to the DNase I enzyme itself, at least under the conditions used to digest chromatin for DNase-seq experiments. This was not seen in previous reports on DNase I digestion of open chromatin [4], [5], although we note that those reports looked only at the gross distribution of signal across broad regions of the genome, rather than the sequences of the individual tags.

### Measuring and Filtering Read-level Bias

In order to gauge the level of bias in each tag, we constructed two position weight matrix (PWM) models, 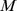 and 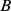, to represent the biased and background sequence patterns respectively, for each sample. The 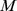 model was generated from positions −5 to +9 (with respect to the 5′ end of tags) of the genome sequence relative to the mapped sequence tags as shown in Figure 1, capturing the majority of the constraint observed. The 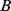 model was constructed similarly, but from flanking sequences offset 100bp from the position of each tag. The two PWMs were then applied to each sequence tag (T) to generate a bias score 
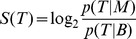
 (i.e. the log ratio of the probability of T being drawn from the biased and background models respectively). The higher the score, the better the fit to the 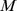 PWM model and the more biased the tag. See the “Position weight matrix models” section in Methods for more details.

**Figure 2:**
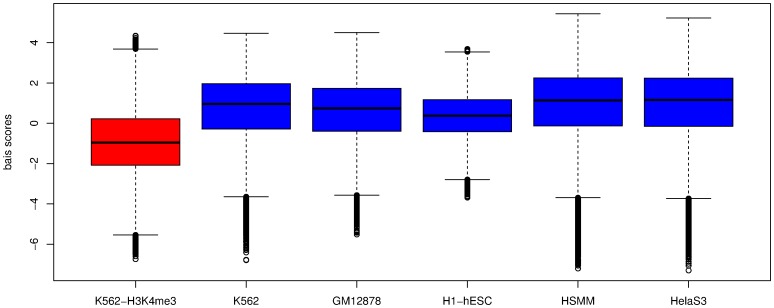
Bias scores for DNase-seq and control reads. Distributions of bias scores (log ratio of M *vs.* B motif scores) for DNase I data from five cell lines analysed in this study(blue) are compared with Histone ChIP-seq as a control set(red).

In our analysis of these five cell types (as described in Table 1 as Main Data), we consistently observed a substantial bias towards positive bias scores. This was quite different to the distribution seen for ChIP-seq reads from the same cell types, which showed a distribution of bias scores towards negative scores (Figure 2). While the range of scores seen is quite similar, the DNase-seq reads are strongly enriched towards the upper end of the scale. We therefore suggest that the probability of DNase I cutting chromatin in a given location depends on both the openness of the chromatin and the sequence at the individual site.

**Figure 3:**
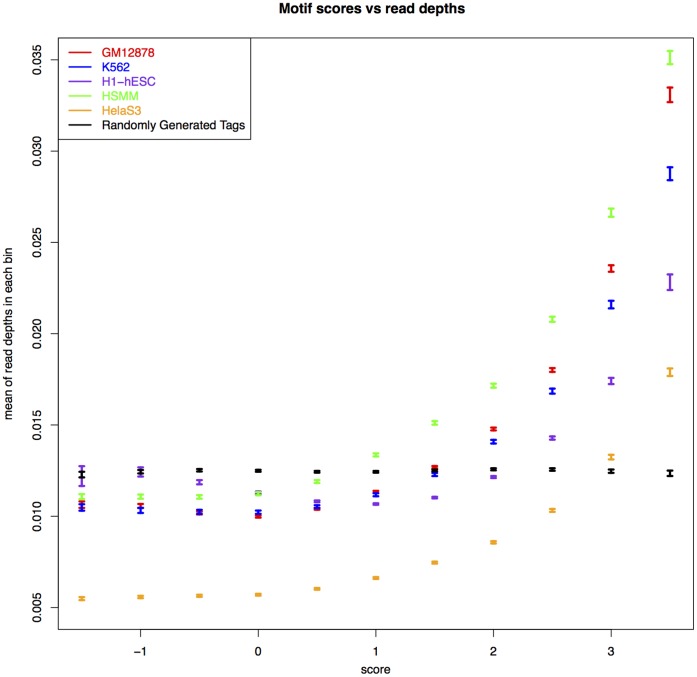
Reads with high motif scores are substantially over-represented. Chromosome-wide motif scores and read depth are calculated at each base pair. The binned motif scores for each cell line (x-axis), mean and standard errors(bars at each point) of read depths at each position (y-axis). An estimation of the expectation is illustrated as the black points which is the result of the same analysis but over a set of randomly picked tags. See Bias scores vs. read depths in Methods for more details.

We then investigated whether calling regions of open chromatin from DNase-seq data could be improved by filtering the set of tags used to generate a set of DHS. From Figure 3, we can surmise that tags with the highest bias scores are substantially over-represented. By reducing the range of bias scores in the data set, we can get closer to a situation whereby the number of DNase-seq tags in a region depends solely on the local chromatin state. In order to find out to what extent removing the bias tags improves the signal detection at the DHS level, we filtered the bias tags with different thresholds (see Methods). We then ran the F-Seq algorithm [12] and obtained a DHS set from each filtered tag set. We measured the overlap of the DHS set with the union of ChIP-seq peak calls from a panel of 13 sets of transcription factors. This set of 13 TFs consists of common TFs in K562, GM12878 and HeLa-S3 and their ChIP-seq data are available (see “How filtering bias tags improves signal detection” in Methods). We take this set as a proxy for active regulatory elements in the genome. Note that ChIP-seq data of these TFs were not available for HSMM and H1-hESC and therefore they were excluded from this overlap analysis. Given the distribution of bias scores, a threshold of 5 means the entire set of DNase-seq tags are included. Thresholds of less than −1 were not used because the resulting filtered tag sets were too small for F-Seq to generate meaningful DHS sets.

**Figure 4:**
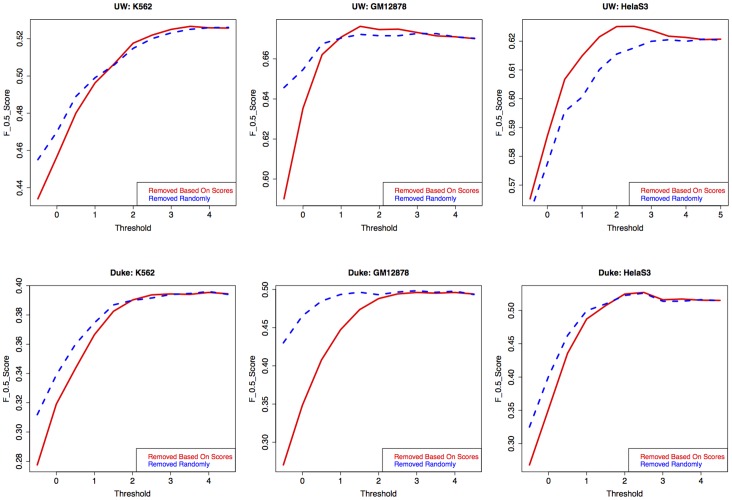
Filtering biased tags improves discrimination. Top panel: Discriminant ability (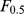 scores) for DHS calls using subsets of the DNase-seq tags (removing tags above each bias score) from UW for three cell lines compared with a union of 13 TFs(blue curve). The red dashed curve is to illustrate if the number of tags removed at each score, has any effect on discriminant scores. For this, the same number of tags at each score are removed and the discriminant scores are measured (red dashed curve). Bottom panel: Illustration of results of the same analysis but for the Duke data.

The result of this analysis is illustrated in Figure 4. In this analysis we started with a threshold equal to the greatest bias score for each cell line then lowered it progressively with a step size of −0.5. We obtained a set of DHS at each threshold and assessed them by measuring the base-level precision and recall compared to the panel of transcription factors (red solid curve in Figure 4). The overlap in this analysis was measured using the 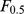 score (see Methods). As we can see from Figure 4, removing the highest scoring (most over-represented) tags improves the F-score for the UW data. In order to evaluate to what extent this improvement is due to DNase I sequence specificity rather than an effect of the number of reads removed from each set, we performed a second round of analysis in which rather than removing tags with a score above a specific threshold, we removed the same number of tags at random. The result of this analysis is shown by the blue dashed curves in Figure 4. We can see that by removing the most biased tags from the UW data we achieve better F-scores than can be achieved by removing tags at random. We also see that by filtering more tags, the discriminant score drops again, probably because so many tags have been excluded that this leads to a greater loss of sensitivity in F-Seq DHS-calling performance than from the further reduction of sequence bias.

These results however do not seem to apply to the Duke data (Figure 4 lower panel) in which the F-score when removing biased tags behaves no better than when removing tags at random. This figure also shows that the F-score for the Duke data is considerably lower than that for the UW data, regardless of threshold. It is not very clear to us why the overlap in the Duke data is lower and/or why removing bias tags does not seem to help. However, as discussed earlier, the bias in the Duke data may be due to a combination of effects from both DNase I and MmeI making our model incapable of discrimination.

## Discussion

Despite the great contribution of high throughput sequencing technologies to our understanding of transcriptional regulation, substantial challenges remain in applying optimal strategies to analyse and understand these data. Different experimental data sets require careful characterization of the biases present to ensure analysis methods are tuned to take best advantage of the data.

Since its discovery over 30 years ago, DNase I has made a major contribution to our understanding of chromatin state and regulatory elements. Over this period, its structure and molecular mechanism have attracted a great deal of research [21]–[25]. One of the key conclusions of these studies is that the cleavage rate of the enzyme is strongly correlated to the width of the minor groove and the stiffness of the DNA. However, to the best of our knowledge, the enzyme’s sequence specificity when cutting genomic targets has not been systematically studied. While two previous studies [4], [5] have claimed that the enzyme has no intrinsic sequence specificity, we do not believe that this represents a comprehensive analysis. Firstly, the reader can only see a screenshot of a part of the genome in [4] but no statistics over the entire genome or an entire chromosome are reported. Secondly any sequence specificity might depend on some other experimental parameter such as temperature, digestion time, concentration of DNase I, salt or buffer composition. Our results show that while the preferred sequence around the DNase cut-site is very consistent for a given protocol, the exact degree of bias varies from sample to sample.

FAIRE-seq (Formaldehyde Assisted Isolation of Regulatory Elements) [20] is another currently used strategy for the detection of open chromatin regions. As the reader can see from Figure S5, the sequence pattern we have described here is not found in FAIRE data because the DNase I enzyme is not used in those experiments. Therefore it is natural to ask whether FAIRE might be an alternative source of unbiased data. However, a recent study by Song et. al. [20] shows rather that FAIRE and DNase I are complementary to each other as FAIRE tends to detect a better signal at distal regulatory elements whereas DNase I tends to behave better in promoter regions.

Given that there is no obvious substitute for DNase I data and the bias we demonstrated it is important that improved analysis methods are developed. The strategy we present for reducing the bias in DNase-seq data sets, by discarding reads derived from the most nearly-optimal cleavage sites, is clearly suboptimal since even the highest-scoring reads are still much more likely to come from regulatory elements and therefore indicate open chromatin – by discarding them, we lose information. However, it serves as a proof-of-concept that software tools aware of DNase bias may be able to generate more accurate sets of DHS calls than existing tools that just rely on read density. One plausible strategy would be to consider the probability – based on sequence – of observing a cut at each position in the genome and using this as the prior for a Bayesian model of the DNase-seq data set.

The NIH Roadmap Epigenomics Consortium [26], [27] has recently proposed to assess the quality of ChIP-Seq and DNase I data and to provide each data set with a quality flag. However, the proposed metrics are based only on the signal-to-noise ratio, *i.e.* the fraction of short read tags that overlap the enriched regions defined by peak calling algorithms. We suggest that while this kind of assessment is important, it may not be sufficient to fully understand the quality of a sequencing data set.

## Methods

### Position Weight Matrix Models

We calculated two position weight matrices (PWM) as follows: PWM M was calculated from a real tag set and called the biased model. The most likely positions for bias are in the range [-5,9] on the 5′ prime side of the tag, so the biased model only considers these 15-mer sequences (note that our coordinate system contains a zero location). In order to estimate the background, PWM B was calculated by shifting real tags 100pb in the 5′ direction. This model was called the background model. Several alternative shifts were tried and the results remained invariant (see Figure S4). One may learn the background model by randomly choosing fixed length tags from the chromosome, however as can be seen from Figure S4 the difference is negligible. In this study we chose to use shifted tags to maintain a chromosomal distribution similar that of the real tags.

PWMs were made based on:
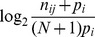
(1)where 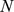 is the number of sequences, 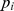 is the expected frequency of 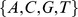 and 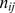 is the frequency of nucleotide 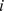 at position 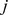. In this study the expected frequencies for 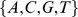 were taken to be 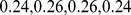 respectively, which is the frequency of nucleotides in the DHSs of the five cell lines studied. Each tag T was then assigned a bias score defined as the log ratio of the probability of that tag being drawn from the biased model to the probability of that tag being drawn from the background model. In other words:
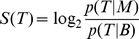
(2)


### Effect of Filtering Bias Tags on Signal Detection

In order to quantify the occupancy level of DHSs at various bias scores with other TFs, we collected a set of 13 TFs for which narrow peak data were available for at least three cell lines GM12878, K562 and HeLa-S3. This set consists of c-Fos, CORESTSC30189, ELK, JunD, MAX, MAZAB85725, NF-YA, NF-YB, NRF1, POL2, RAD21, SMC3, TBP, and USF2. The narrow peaks of these TFs were downloaded from http://hgdownload.cse.ucsc.edu/goldenPath/hg19/encodeDCC/wgEncodeSydhTfbs. The union of the narrow peaks of these 13 TFs was considered as the gold standard set. The precision was defined as 
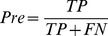
 and similarly the recall defined as 
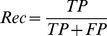
. The overlap and/or discriminant score was defined as:
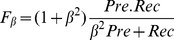
(3)We note that the recall scores will be conservative estimates since not every regulatory region will be bound by one of the factors in this panel. For this reason we have set 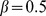.

We calculated the bias scores of K562, GM12878 and HeLa-S3 DNase-seq reads as described previously. These scores ranged from −7 to 5. Starting from the maximum bias score of each set *i.e.* the complete set and finishing at threshold −1, we iteratively ran the F-Seq algorithm, obtained a set of DHS calls and then reduced the threshold by 0.5 units to exclude the next most high-scoring tags. For thresholds less than −1, there were insufficient tags to get meaningful results from F-Seq, therefore thresholds less than −1 were excluded from our analysis.

### Bias Scores Versus Read Depths

For each of the five cells lines a PWM was constructed as described above. One PWM was also constructed using randomly picked tags(the same number as the real tags) to reflect the background expectation. Then, for each of these PWMs the chromosome was scanned and a motif score calculated for each position for each of the strands. The maximum of these two scores was considered as the motif score at the given position. The read depth at each position was also counted. The motif scores were binned (with a bin width of 0.5 bits), and the mean and standard error of the read depth for sites within each bin were calculated.

Raw data used for this study is available at http://genome.ucsc.edu/ENCODE/downloads.html under the options “UW DNaseI HS” and “Duke DNaseI HS”, and “SYDH TFBS” for the transcription factors. For more details of the data sets used the reader is referred to Table 1.

## Supporting Information

Figure S1 **Sequence around the 5′ ends of DNase-seq reads from other labs.** Additional data sets following the analysis described in Figure 1.
(TIF)

Figure S2 **Variation in GC content.** Variation in GC content across 5 cell lines for the UW and Duke data sets.
(TIF)

Figure S3 **Enrichment of motifs in open chromatin regions.** Illustrated here are motif scores in DHSs regions (red) and in the same number of randomly picked sequences(blue). As we can see, the distributions for each cell line are almost identical suggesting that motifs are not substantially enriched in open chromatin.
(TIF)

Figure S4 **Effect of shifting parameter.** Different shifting lengths were applied to show that the background model is not strongly dependent on shifting lengths. The shifting lengths applied were 40, 60, 80, 100, 200bp and also the last histogram illustrates the scores from randomly picked tags rather than shifting. As we can see the distribution of scores are very similar.
(TIF)

Figure S5 **No sequence pattern in FAIRE data.** Illustrated here is the alignment of short read tags (plus 10bp offset from each end) from FAIRE data for GM12878 cell line. This figure illustrates only short read tags over chromosome 22. This data set is available in http://hgdownload.cse.ucsc.edu/goldenPath/hg19/encodeDCC/wgEncodeOpenChromFaire/.
(TIF)
