# Supplementary figures and images for: Chromatin Accessibility Data Sets Show Bias Due to Sequence Specificity of the DNase I Enzyme
Source: PLoS One. 2013 Jul 26;8(7):e69853. doi: 10.1371/journal.pone.0069853 (PMC3724795; doi:10.1371/journal.pone.0069853)

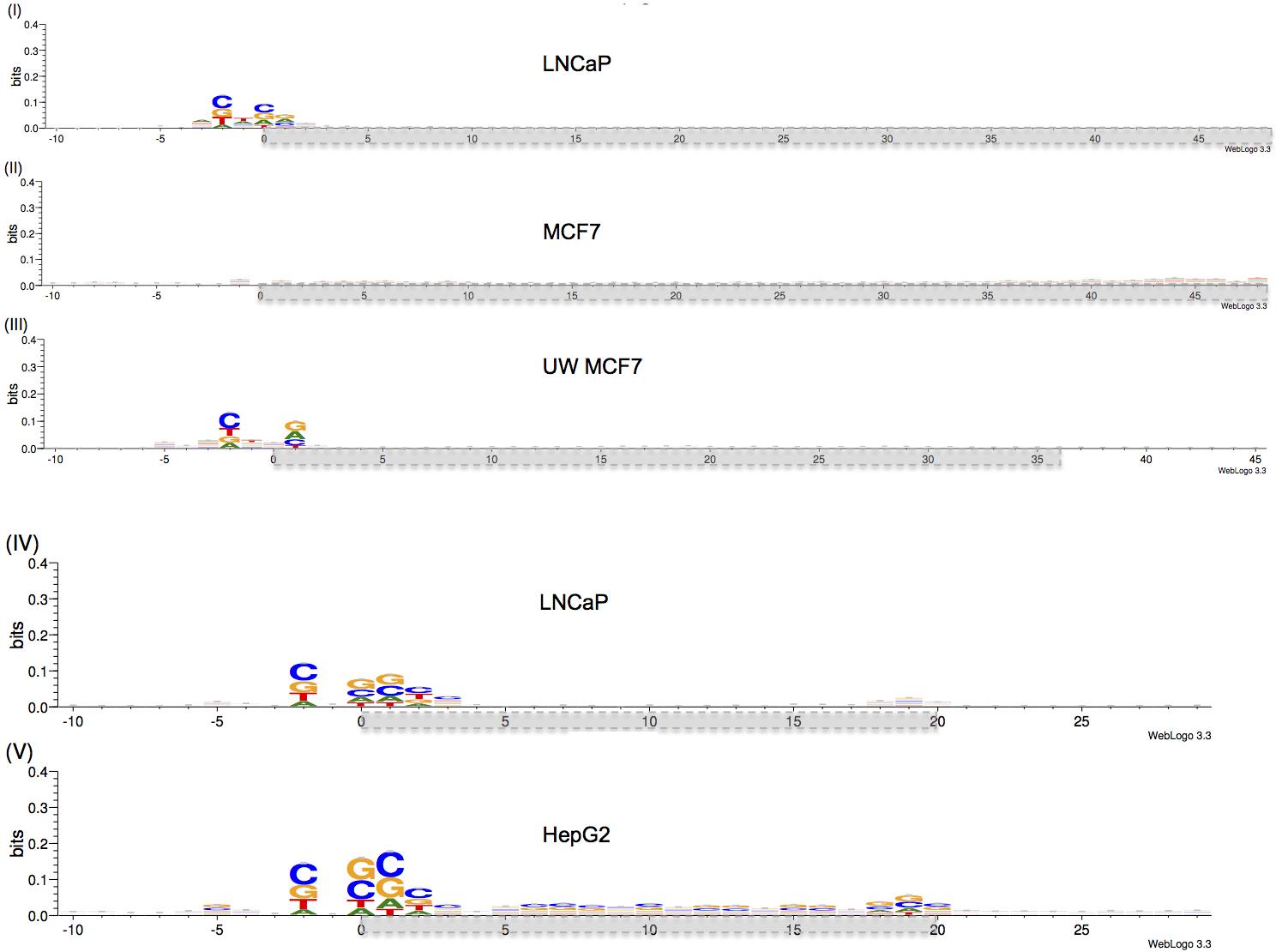

Supplement: Figure S1 — Sequence around the 5′ ends of DNase-seq reads from other labs. Additional data sets following the analysis described in Figure 1. (TIF) [file pone.0069853.s001.tif]

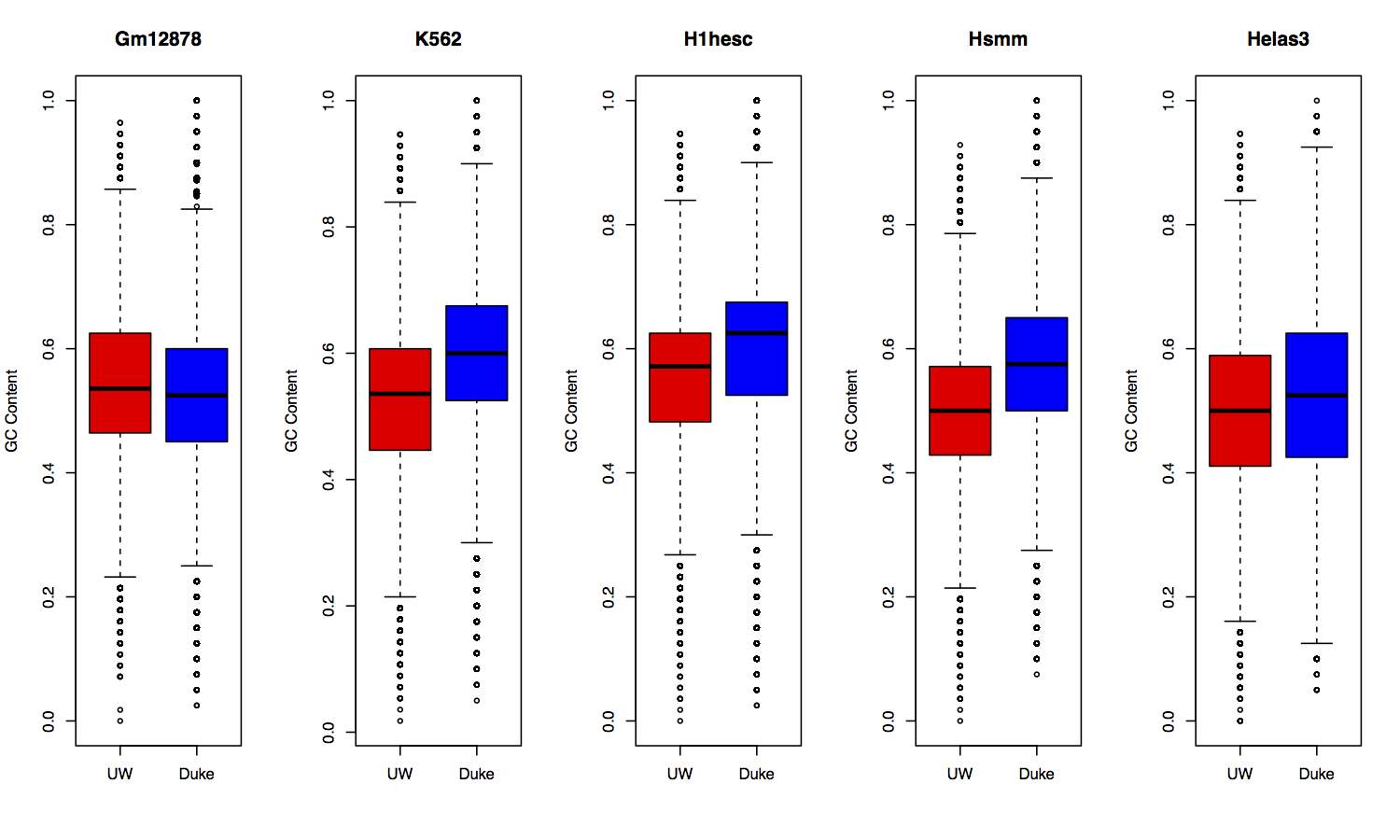

Supplement: Figure S2 — Variation in GC content. Variation in GC content across 5 cell lines for the UW and Duke data sets. (TIF) [file pone.0069853.s002.tif]

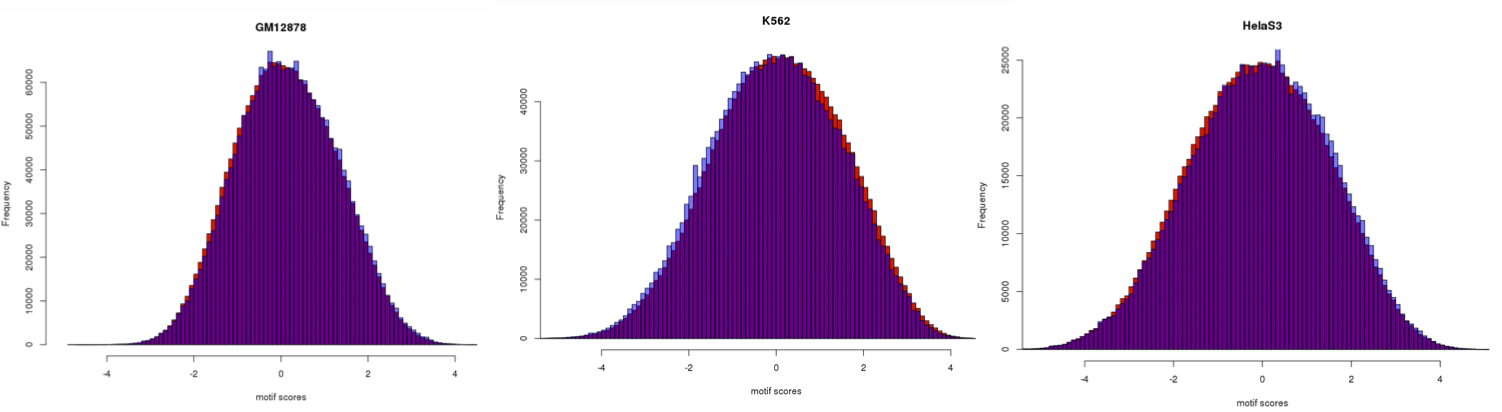

Supplement: Figure S3 — Enrichment of motifs in open chromatin regions. Illustrated here are motif scores in DHSs regions (red) and in the same number of randomly picked sequences(blue). As we can see, the distributions for each cell line are almost identical suggesting that motifs are not substantially enriched in open chromatin. (TIF) [file pone.0069853.s003.tif]

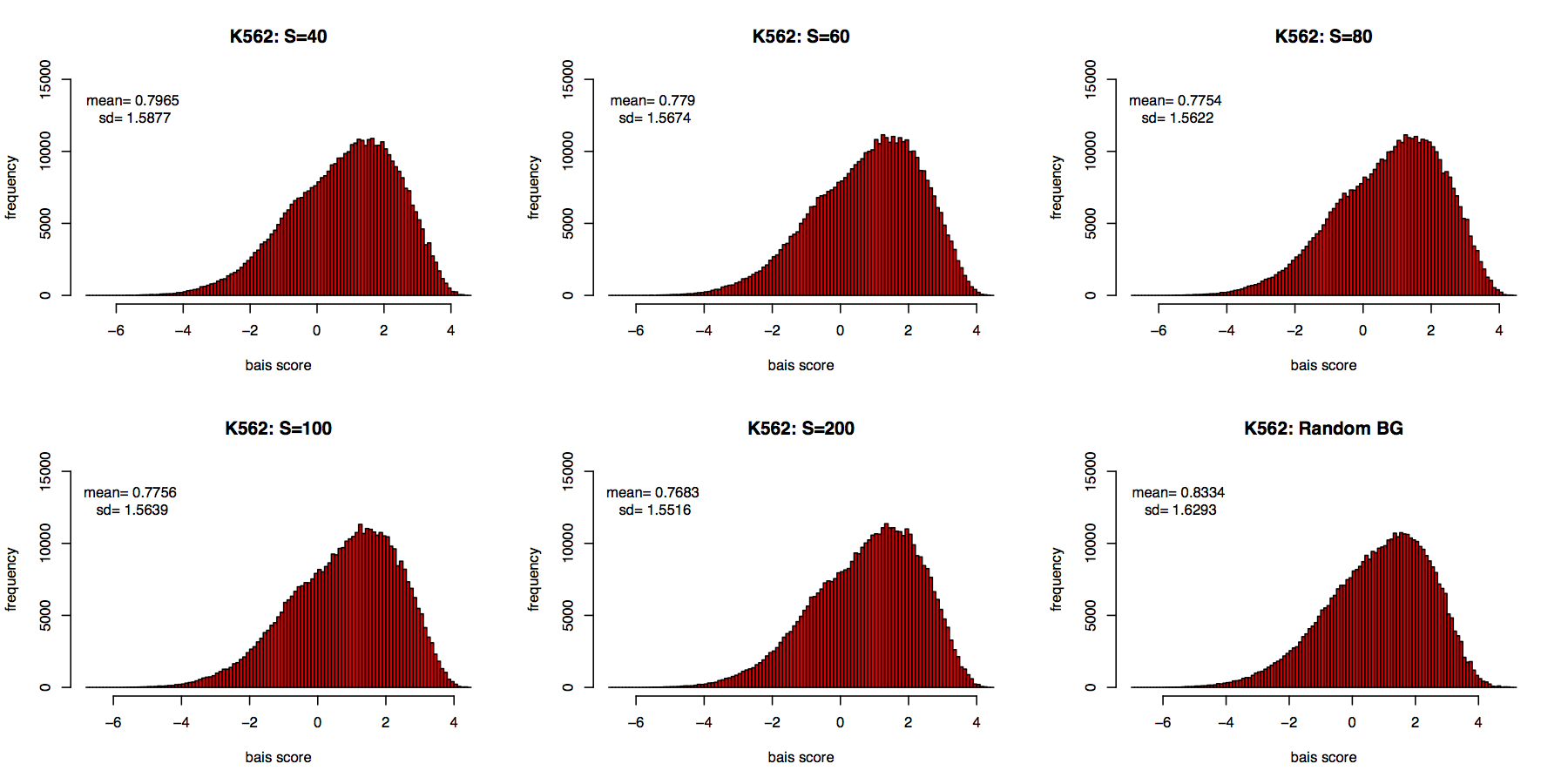

Supplement: Figure S4 — Effect of shifting parameter. Different shifting lengths were applied to show that the background model is not strongly dependent on shifting lengths. The shifting lengths applied were 40, 60, 80, 100, 200bp and also the last histogram illustrates the scores from randomly picked tags rather than shifting. As we can see the distribution of scores are very similar. (TIF) [file pone.0069853.s004.tif]

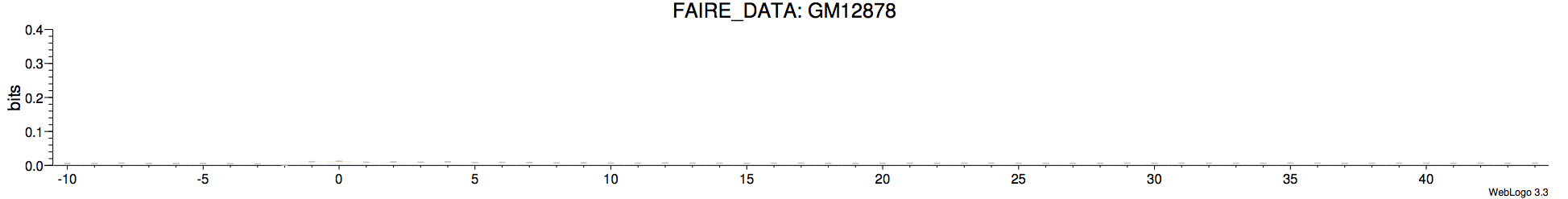

Supplement: Figure S5 — No sequence pattern in FAIRE data. Illustrated here is the alignment of short read tags (plus 10bp offset from each end) from FAIRE data for GM12878 cell line. This figure illustrates only short read tags over chromosome 22. This data set is available in http://hgdownload.cse.ucsc.edu/goldenPath/hg19/encodeDCC/wgEncodeOpenChromFaire/. (TIF) [file pone.0069853.s005.tif]
